# Evidence from 2024 Storm Boris indicates anthropogenic climate change amplifies floods more than extreme precipitation

**DOI:** 10.1126/sciadv.aef7703

**Published:** 2026-10-28

**Authors:** Peter Miersch, István Dunkl, Antonio Sánchez-Benítez, Marylou Athanase, Oldrich Rakovec, Daniel Klotz, Sebastian Sippel, Helge Goessling, Jakob Zscheischler

**Affiliations:** ^1^ Department of Compound Environmental Risks, Helmholtz Centre for Environmental Research – UFZ, Leipzig, Germany.; ^2^ Department of Hydro Sciences, TUD Dresden University of Technology, Dresden, Germany.; ^3^ Leipzig Institute for Meteorology, Leipzig University, Leipzig, Germany.; ^4^ Alfred Wegener Institute, Helmholtz Centre for Polar and Marine Research, Bremerhaven, Germany.; ^5^ Faculty of Environmental Sciences, Czech University of Life Sciences Prague, Prague, Czechia.; ^6^ Interdisciplinary Transformation University Austria, Linz, Austria.; ^7^ Google Research, Vienna, Austria.

## Abstract

Anthropogenic climate change intensifies extreme precipitation, but how it affects flood hazards remains uncertain. In September 2024, Storm Boris triggered catastrophic flooding across central Europe, underscoring the need to quantify the contribution of human-caused climate change to flood intensity for effective risk management. Using a storyline approach, we show that precipitation associated with Storm Boris would have been 6 to 9% lower in the absence of anthropogenic climate change, whereas flood peaks would, on average, have been reduced by 14 to 22%. Our estimates are based on nudged simulations from two climate models coupled with two hydrological models across hundreds of affected catchments. The results imply that anthropogenic forcing amplified flood peaks by a factor of 2.1 to 2.9 relative to the increase in event precipitation. Overall, we demonstrate that attributing precipitation alone would substantially underestimate flood hazard amplification from climate change—an effect that should inform future risk assessments and loss estimates.

## INTRODUCTION

Floods are among the most impactful climate-related disasters globally (*1*). The flooding across central Europe associated with the 2024 Storm Boris caused widespread damage, evacuations, and 27 fatalities (*2*), raising questions about the role of anthropogenic climate change in such events. Rain gauge observations show that heavy precipitation events are becoming more frequent across Europe (*3*, *4*). This trend is consistent with physical understanding that warming increases atmospheric moisture content, intensifying precipitation extremes with high confidence (*5*). In contrast, detecting long-term trends in flood magnitude remains challenging due to confounding factors and limited record lengths (*6*, *7*). Extreme event attribution offers an alternative to trend analysis by isolating the contribution of anthropogenic climate change to individual extreme events and has emerged as a critical tool for quantifying how it affects their magnitude and frequency (*8*–*10*), which is essential to inform adaptation strategies and risk assessments under changing climatic conditions (*11*).

It has already been shown that anthropogenic climate change increased the intensity of extreme precipitation associated with Storm Boris by between 7% (*12*) and 9% (*13*) and doubled the likelihood of the event (*12*). However, the complex response of floods to changes in precipitation could imply that modest precipitation intensification can trigger disproportionate increases in flood magnitude (*14*). This arises from threshold-dependent runoff generation, antecedent soil moisture conditions, and catchment storage dynamics (*15*–*17*). Consequently, attributing only extreme precipitation events to anthropogenic climate change does not adequately capture its effects on flood magnitudes. Although the nonlinear hydrological response to precipitation changes is well established in principle (*15*, *16*), event attribution studies overwhelmingly focus on precipitation effects only (*14*), and the reported numbers may plausibly serve as de facto proxies for flood hazard change when results are communicated to nonspecialist audiences and in risk assessment contexts.

Here, we use storylines—physically self-consistent alternative realizations of observed events (*18*)—to quantify the role of anthropogenic climate change in both precipitation and flood magnitude in hundreds of river catchments affected by Storm Boris. This is achieved by forcing two hydrological models with observed and counterfactual meteorological inputs, where the latter are generated through nudged climate model simulations. These simulations offer advantages over using traditional free-running climate model ensembles by maintaining the exact dynamical (atmospheric circulation) conditions that produced the observed event, enabling robust quantification of the thermodynamic climate change effects (*19*). In particular, this conditional attribution approach acknowledges that extreme events require unusual meteorological conditions and can be amplified by climate change. Although dynamical changes constitute a major source of uncertainty in future projections (*20*–*22*), thermodynamic changes have dominated historical multiday extreme precipitation changes in our central European study region (*23*). Using nudged climate model simulations we isolate the thermodynamic component of climate change (*24*). This storyline approach, which is strongly conditioned to the event dynamics, differs fundamentally from unconditional probabilistic attribution (*9*, *10*), which estimates how climate change altered the probability or intensity of a class of events rather than the specific observed event. The meteorological changes derived from probabilistic attribution cannot be directly transferred to a hydrological model simulating a specific event.

Our study contains three major innovations. First, although multimodel ensembles are standard in attribution analyses based on free-running climate simulations (*9*), storyline approaches with nudged climate models thus far typically use only one climate model (*13*, *25*); here we advance the methodology by using a two-model ensemble to characterize structural climate model uncertainty within this emerging framework. Second, we directly attribute flood peak magnitude rather than precipitation extremes, quantifying the effect of anthropogenic climate change on impact-relevant hazards. Third, we use two structurally distinct hydrological models to simulate hundreds of river catchments, addressing uncertainty arising from different representations of hydrological processes in translating precipitation changes into flood responses.

## RESULTS

### Anthropogenic climate change amplified precipitation during Storm Boris

The extratropical cyclone named Storm Boris (*26*) led to extreme precipitation over Austria, Czechia, and Poland, locally exceeding 400 mm of accumulated precipitation in just 5 days (*27*). Subsequent flooding prompted emergency declarations and activation of Copernicus Emergency Management Service On-Demand Rapid Mapping (EMSR) (*28*) in Austria (EMSR756 and EMSR764), Poland and Czechia (EMSR756), as well as Germany, Slovakia, Romania, and Hungary (EMSR755, EMSR757, and EMSR759) (*2*). The event is well captured in precipitation observations, like the gridded E-OBS dataset (*29*) (v31.0e), with extreme precipitation clusters over Lower Austria and the Czech and Polish border regions reporting accumulated precipitation up to 398 mm (Fig. 1A). Runoff reconstructions of the flood peaks (*30*, *31*) indicate that 484 catchments exceeded moderate 25-year return period floods, with 103 catchments surpassing extreme 200-year return periods (Fig. 1B) (Materials and Methods). Storyline attribution with nudged climate model simulations of the event with AWI-CM-1 (AWI Climate Model 1) (*13*) and CESM2 (Community Earth System Model 2) (*32*, *33*) indicates that anthropogenic climate change contributed to enhanced extreme precipitation during the event through thermodynamic effects (Materials and Methods). Simulations with preindustrial greenhouse gas and aerosol concentrations show 9.3 and 6.4% lower accumulated precipitation over the event region in CESM2 and AWI-CM-1, respectively, compared to simulations with observed emissions (factual conditions; Fig. 1, C and D). The relative precipitation difference between factual and counterfactual climate conditions is relatively uniform spatially in AWI-CM-1, whereas precipitation differences are larger in the west of the study region in CESM2 compared to the east, with both models capturing the precipitation event well (figs. S1 and S2).

**Fig. 1.**
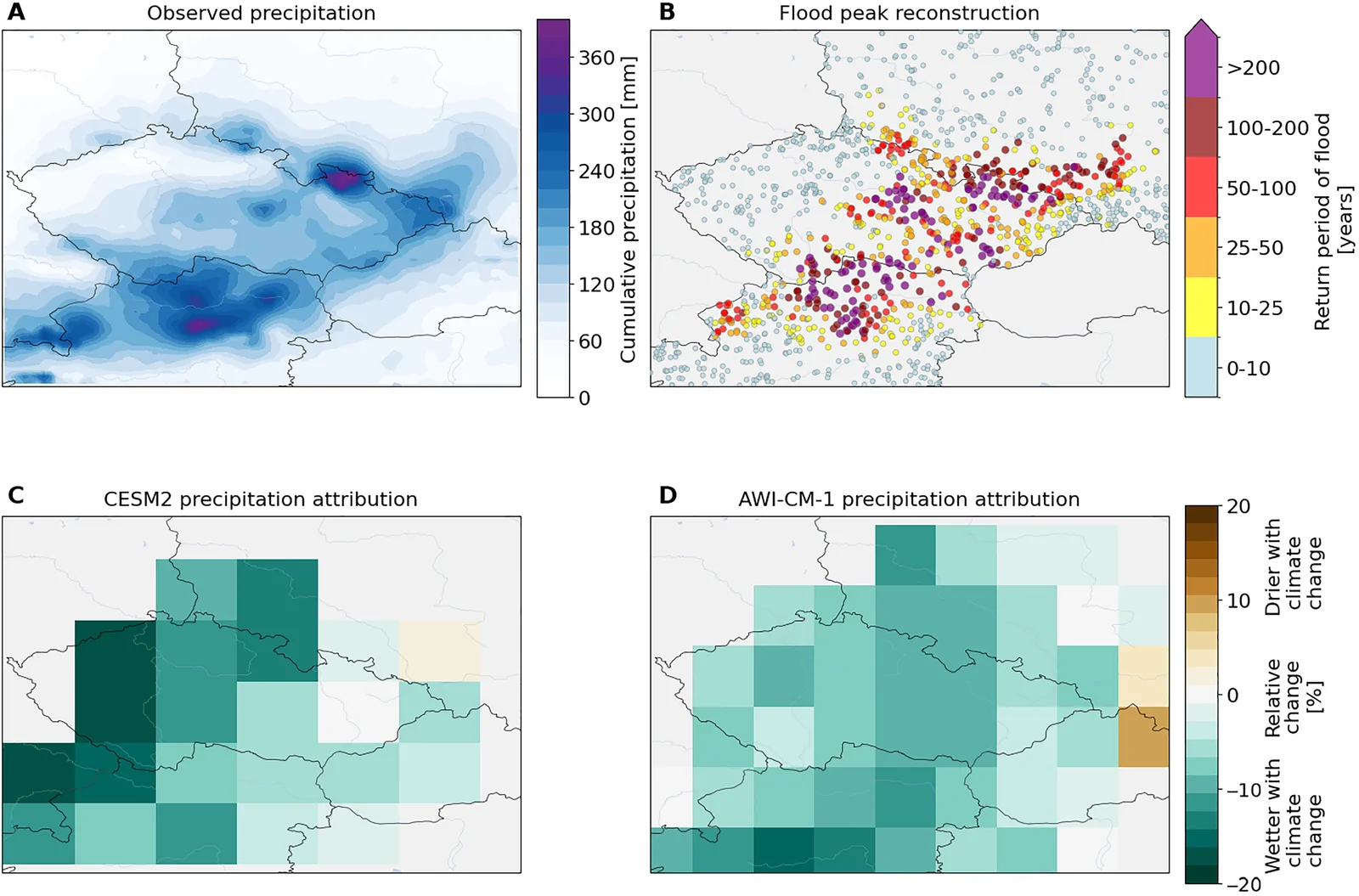
Precipitation associated with Storm Boris led to severe flooding and was amplified by anthropogenic climate change. The event region used is 12°E to 20°E and 47°N to 52.5°N with event duration from 12 to 16 September 2024. (**A**) Observed cumulative precipitation (*29*) at 0.1° resolution. Spatial average: 105 mm; maximum: 398 mm. (**B**) Return periods for reconstructed runoff peaks [based on the EARLS model (*30**,* *31*); Materials and Methods] for 1397 catchments. Flood peaks have return periods above 25 years in 484 catchments (threshold used for later analysis) and above 200 years in 103 catchments. (**C**) Relative precipitation change between counterfactual simulation with preindustrial greenhouse gas concentrations and factual simulation with observed 2024 greenhouse gas concentrations for the CESM2 climate model. Accumulated precipitation in the counterfactual is 9.3% lower than in the factual, averaged over the event region. (**D**) Same as (C), but for the ensemble mean of the AWI-CM-1 model. The average accumulated precipitation is 6.4% lower.

The identified changes are in line with expected Clausius-Clapeyron scaling, observed daily extreme precipitation intensification, and attribution studies of the same event. This relationship describes the water holding capacity of the atmosphere as a function of temperature at ∼7% per degree warming (*34*) and explains observed extreme precipitation intensification, in particular for long-duration (≥1 day) events (*22*, *24*). Beyond that, stronger vertical air ascent driven by higher temperatures may have further amplified the most extreme precipitation (*13*). Our results are consistent with these previous attribution studies: A World Weather Attribution study found a 7% precipitation increase in the observed event compared to preindustrial conditions (*12*). Using storyline attribution with AWI-CM-1, a previous work with less stringent nudging than what is used in the present study found a 9% increase relative to a counterfactual climate and an eastward displacement of precipitation (*13*), potentially reflecting weaker wind constraints than applied here. Note that event region and duration definitions differ between studies. In addition, both studies use the counterfactual as reference, whereas we use factual simulations as reference. This choice is intentional: The factual event represents what was experienced and is thus more relevant for attributing hazards such as floods and droughts (*35*). Furthermore, for attributing flood peak changes, our experimental design provides higher confidence in the factual hydrological simulations derived from meteorological observations than counterfactual hydrological simulations based on storylines, supporting the use of factual simulations as reference (Materials and Methods).

### Anthropogenic climate change amplified flooding more than precipitation

Precipitation attribution alone inadequately captures anthropogenic climate change effects on river flood hazards (*14*). High-resolution hydrological models enable explicit flood peak simulations and, when coupled with storylines from nudged climate models, permit direct flood event attribution. Given the coarse resolution of the climate models used here (∼1°), and their biases even after nudging, we create counterfactual forcing for the hydrological models by applying the relative difference in precipitation and absolute difference in temperature between the counterfactual and factual climate model simulations (Fig. 1, C and D; sometimes called the “delta approach”) to the higher-resolution gridded observation-based precipitation and temperature (Fig. 1A) for the event duration [12 to 16 September 2024; Materials and Methods]. Consequently, we have a single factual hydrological simulation (the unaltered meteorological observations), one counterfactual hydrological simulation from the CESM2 model, and 25 counterfactual hydrological simulations from the AWI-CM-1 model ensemble members (Materials and Methods). As the AWI-CM-1 ensemble members show very little variation between them, we only report ensemble mean results in the main text. For these counterfactual hydrological simulations, we modify precipitation and temperature only during the event itself, thereby preserving the observed antecedent conditions, including pre-event soil moisture states and any storage excess that had accumulated before Storm Boris, identically across factual and counterfactual simulations. This design ensures that flood peaks reflect the full complexity of hydrological processes through which rainfall is translated into runoff, encompassing threshold-dependent infiltration and soil moisture–driven runoff generation. To model the flood peaks, we use two structurally different hydrological models (Materials and Methods). The NeuralHydrology machine learning model (*31*), trained and validated on >5000 catchments (*36*) for the European Aggregated Reconstructions for Large-sample Studies (EARLS) (*30*), simulates runoff as a lumped model for 1397 catchments in our study region. In contrast, the mesoscale Hydrologic Model (mHM) (*37*–*39*) routes the incoming precipitation as runoff through a physics-based simulated river network at a 0.125° resolution.

We first demonstrate our attribution approach through hydrological simulations at the Nysa station on the Nysa Kłodzka river as a case study. At this location, where severe flooding caused extensive damage (*2*) (EMSR756), simulations show substantially reduced flood hazard in the counterfactual hydrological simulations. The flood peak would have been between 10.6% (CESM2) and 12.0% (AWI-CM-1) lower without anthropogenic climate change simulated with EARLS (Fig. 2A). This reduction mirrors the precipitation differences between factual and counterfactual simulations (Fig. 1, C and D), with AWI-CM-1 showing slightly higher sensitivity to warming than CESM2 for this catchment. This is also apparent when comparing the catchment-averaged precipitation, where we observe a decrease between 7.8% (CESM2) and 8.6% (AWI-CM-1) (Fig. 2D). Here, it already becomes apparent that just analyzing changes in precipitation is not sufficient to characterize changes in the flood hazard: The relative change in the flood peak is 1.4 times the change we observe in precipitation for both CESM2 and AWI-CM-1. Similarly, for runoff simulated with the mHM, we find that the flood peak would have been between 10.5% (CESM2) and 13.4% (AWI-CM-1) lower without anthropogenic climate change (Fig. 2B), whereas the accumulated precipitation over the upstream catchment was only between 7.0% (CESM2) and 8.5% (AWI-CM-1) lower (Fig. 2E). This corresponds to a 1.5 (CESM2) to 1.6 (AWI-CM-1) times relative change in the flood peak compared to the relative precipitation change.

**Fig. 2.**
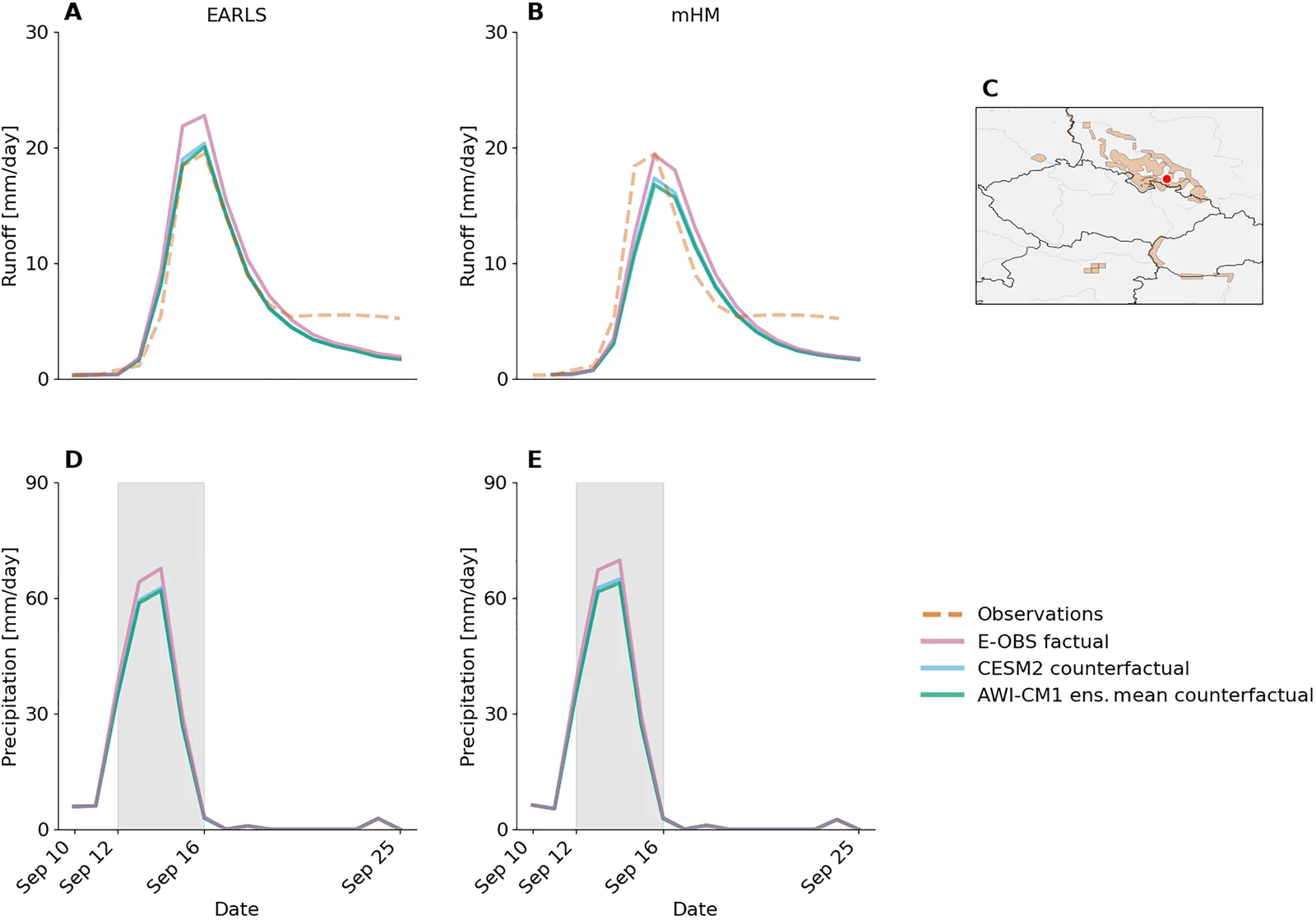
Flooding was amplified by anthropogenic climate change in a highly affected catchment. Results are shown for the Nysa station on the Nysa Kłodzka river, Poland, where severe flooding occurred (*2*). (**A**) Observed streamflow (*36*) and EARLS-simulated runoff. Factual hydrological simulation flood peak: 22.8 mm/day; counterfactual reductions of 10.6% (CESM2) and 12.0% (AWI-CM-1 mean). Observed streamflow (*62*) shown for reference. (**B**) mHM-simulated runoff at the nearest grid cell. Factual hydrological simulation flood peak: 19.4 mm/day; counterfactual reductions of 10.5% (CESM2) and 13.4% (AWI-CM-1 mean). (**C**) Location of the gauging station/grid cell (red dot) and Copernicus EMS rapid mapping areas for Storm Boris flooding. (**D**) Catchment-averaged daily precipitation for EARLS (shaded area: event duration). Accumulated event precipitation: 202.4 mm; counterfactual reductions of 7.8% (CESM2) and 8.6% (AWI-CM-1 mean). (**E**) As (D), but for the mHM upstream grid cells. Accumulated event precipitation: 208.4 mm; counterfactual reductions of 7.0% (CESM2) and 8.5% (AWI-CM-1 mean).

A regional analysis across central Europe confirms the catchment-scale changes but also reveals spatial heterogeneity in flood peak reductions in the counterfactual hydrological simulations as well as differences between climate models (Fig. 3). CESM2 shows reductions in peak runoff of 19.7% in EARLS and 14.4% in the mHM averaged over all catchments experiencing floods with at least a 25-year return period in the factual hydrological simulation (Materials and Methods). AWI-CM-1 exhibits slightly larger reductions of 22.4 and 17.6%, respectively. This is despite the overall smaller difference in event precipitation compared to CESM2 (Fig. 1, C and D) as the reductions in extreme precipitation are larger over affected regions in Czechia and Austria. Consistent with the changes in precipitation patterns (Fig. 1), hydrological simulations derived from CESM2 show the strongest decreases in western regions, particularly the Upper Austria–Lower Austria border, the Bohemia-Moravia border in Czechia, and Poland’s Dolnośląskie province (Fig. 3, A and C). Eastern areas show only small changes or even slight increases in the counterfactual simulations. Hydrological simulations derived from AWI-CM-1 exhibit a more uniform reduction of flood peaks. For both hydrological models, spatial distribution influences catchment averages (EARLS) and grid cell averages (mHM). Simulations for catchments with EARLS are based on gauging stations (*36*) and lack independence due to nesting. For the mHM, grid cells are uniformly distributed but spatially dependent through water routing. Both hydrological models demonstrate similar spatial patterns and flood peak reductions, although the mHM simulates smaller flood peak changes than EARLS (fig. S3). The consistency from both the climate models and the hydrological models suggests high confidence in the robustness of the flood peak attribution signal.

**Fig. 3.**
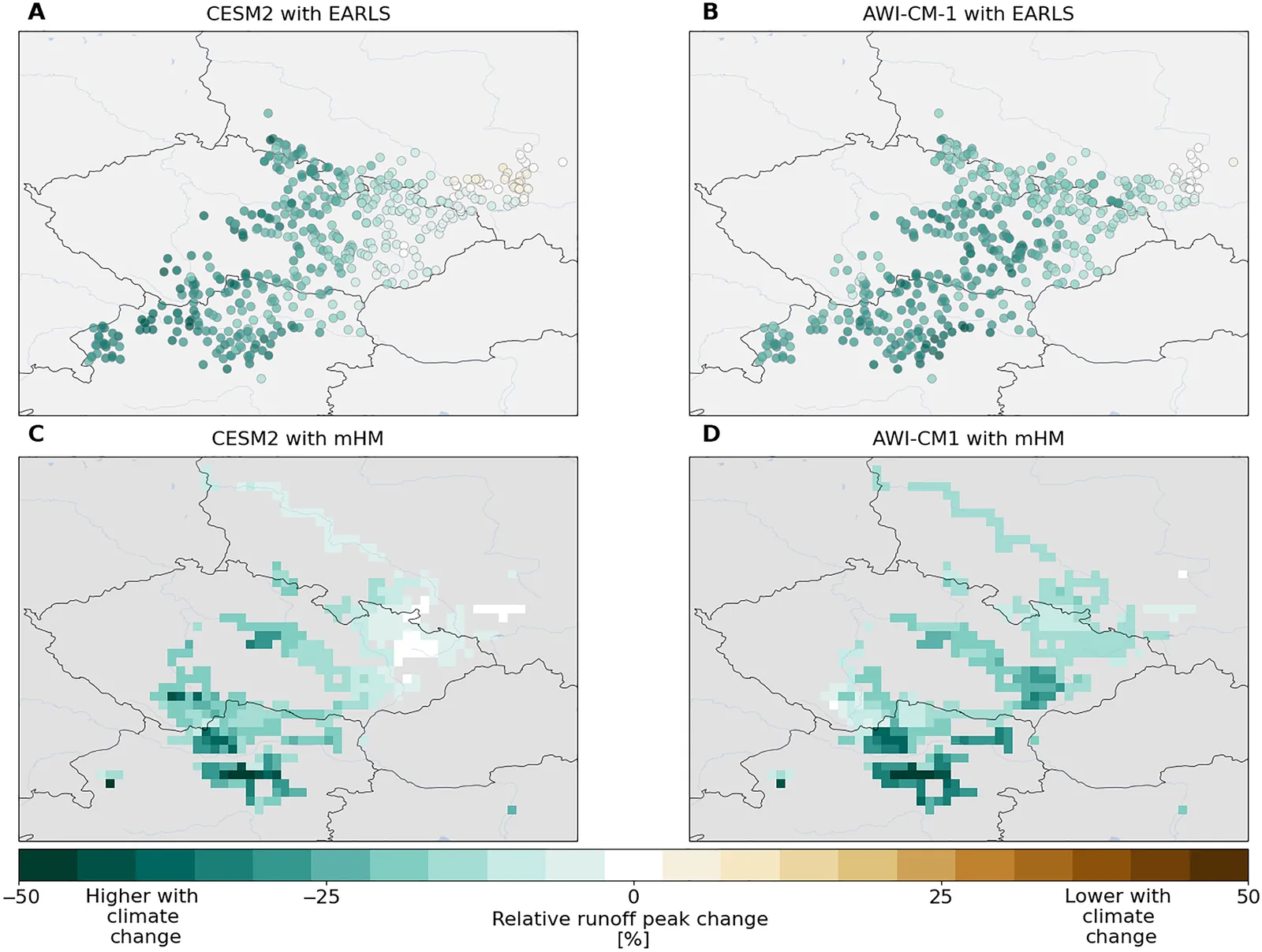
Flooding was amplified across most of the affected region. Relative change in flood peaks between factual and counterfactual hydrological simulations, shown for gauging stations (EARLS; top row) and grid cells (mHM; bottom row) where flood peaks exceeded the 25-year return period in the factual simulation. Negative values indicate reduced flood peaks in the counterfactual without anthropogenic emissions. (**A**) CESM2 counterfactual with EARLS hydrological simulations. Mean reduction: 19.7%. (**B**) Ensemble mean of AWI-CM-1 counterfactuals with EARLS hydrological simulations. Mean reduction: 22.4%. (**C**) CESM2 counterfactual with mHM hydrological simulations. Mean reduction: 14.4%. (**D**) Ensemble mean of AWI-CM-1 with mHM hydrological simulations. Mean reduction: 17.6%.

### Flood intensity amplification outpaces precipitation amplification

Across all model combinations, flood peaks increase 2.1 to 2.9 times as much as event precipitation (Fig. 4). For locations experiencing floods with ≥25-year return periods and ≥5-mm difference in event precipitation between factual and counterfactual climate model simulations, flood peak changes in EARLS are 1.3 to 4.8 times as large as the relative changes in event precipitation, with a mean scaling across catchments of 2.8 for hydrological simulations derived from CESM2 and 2.9 for AWI-CM-1 (Fig. 4, A and B). For the mHM, the scaling factor ranges from −0.4 to 5.8 relative to precipitation, with a mean scaling across grid cells of 2.1 for CESM2 and 2.2 for AWI-CM-1 (Fig. 4, C and D). The stronger scaling in EARLS compared to the mHM reflects differences in model structure and spatial scale: EARLS is a lumped machine learning model applied to mostly small catchments, whereas the mHM is a gridded process-based model operating on larger spatial scales. These scaling factors exceed those reported in previous studies (*40*, *41*), particularly for EARLS, largely because we focus on more extreme flooding events in small catchments.

**Fig. 4.**
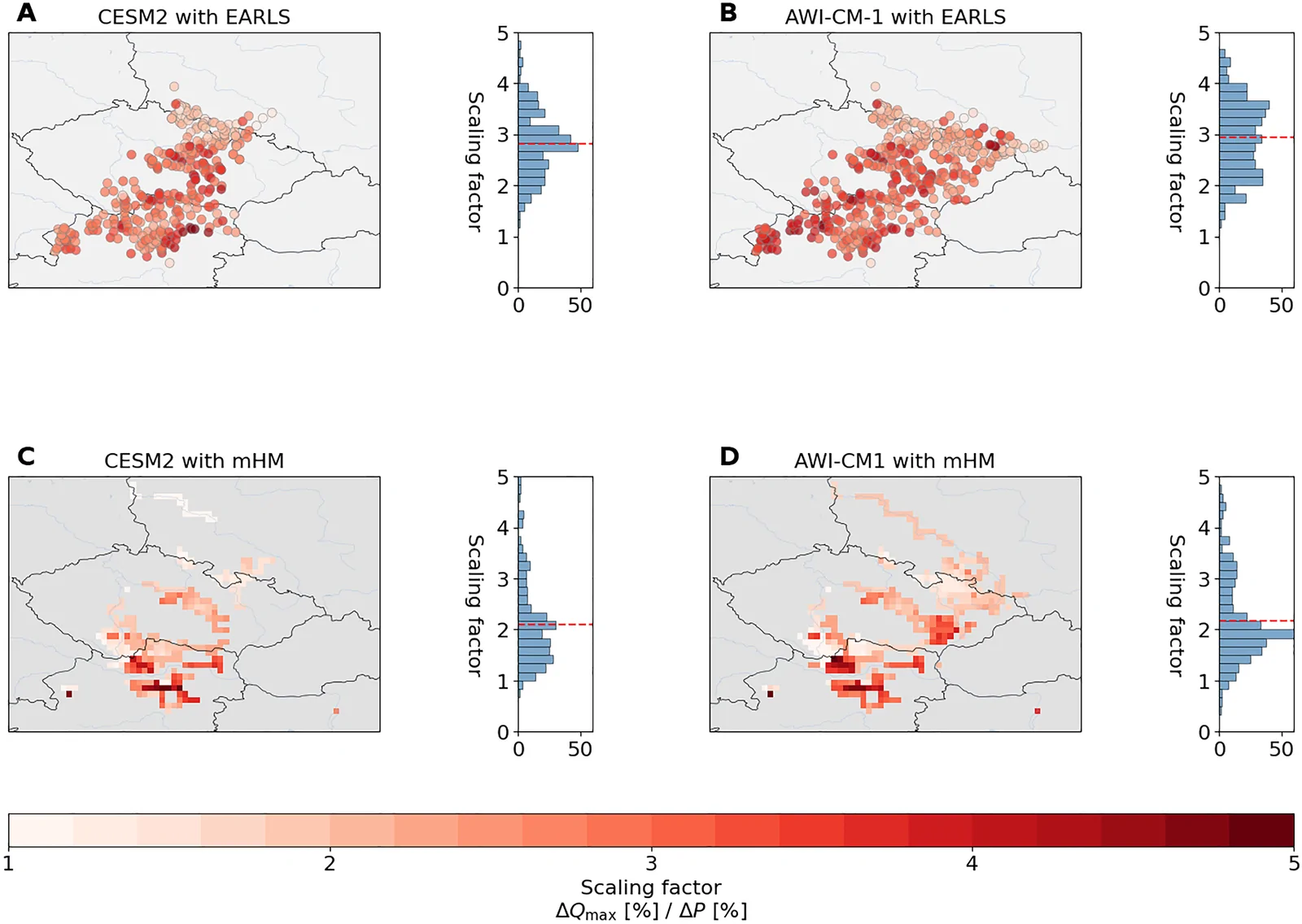
Flood intensity amplified more than precipitation intensity. Scaling factor between relative changes in flood peak (peak runoff Δ*Q*_max_) and relative changes in upstream-averaged event precipitation during the event (Δ*P*) for catchments with ≥25-year return period of the flood peak in the factual hydrological simulations and ≥5-mm difference in event precipitation between factual and counterfactual climate model simulations. (**A**) Scaling factor of hydrological simulation with EARLS and CESM2. Mean scaling factor (red line in the histogram) is 2.8. (**B**) Scaling factor of hydrological simulation with EARLS and AWI-CM-1. Mean scaling factor is 2.9. (**C**) Scaling factor of hydrological simulation with the mHM and CESM2. Mean scaling factor is 2.1. (**D**) Scaling factor of hydrological simulation with the mHM and AWI-CM-1. Mean scaling factor is 2.2.

This amplification occurs because we focus on extreme flooding at the catchment level (return periods above 25 to 50 years), which is highly sensitive to changes in precipitation intensity (*16*). For less extreme floods, soil moisture deficits, which can be amplified through preconditioning by a warmer climate not considered in this study, can dampen flood responses (*15*). For the extreme flooding considered here, anthropogenic climate change amplifies flood hazard more than precipitation changes alone would suggest. This intuition is supported by analysis of the precipitation that is converted to runoff during the event (that is, the runoff coefficient; fig. S4) and the precipitation that is converted to peak runoff (peak runoff coefficient; fig. S5). Not only does the lower event precipitation without climate change directly lead to lower runoff peaks, but the precipitation is also less efficiently converted to runoff peaks (figs. S6 and S7).

The much stronger relative increase in runoff compared to precipitation identified in our study is consistent with previous storyline analyses (*41*) and analyses of observational data (*40*) of other events, and aligns with expectations that flood magnitudes scale directly with precipitation for the most extreme events (*15*, *16*). An amplified response for high flow percentiles consistent with these findings has also been observed in US catchments during summer (*42*).

## DISCUSSION

Using a storyline attribution approach, our study reveals that anthropogenic climate change substantially amplified the flooding associated with Storm Boris beyond what would be expected when studying precipitation intensification alone. The regionally 6.4 to 9.3% decreased precipitation derived from simulations with removed anthropogenic climate change translates to 14.4 to 22.4% reduced flood peaks averaged over all affected catchments. These findings likely extend beyond Storm Boris to other flood events in similar hydroclimatic settings. Although the specific scaling between precipitation and flood magnitude depends on the precipitation event and local catchment properties, the fundamental principle that flood event attribution strongly diverges from precipitation event attribution applies more broadly. Climate change mitigation would therefore substantially reduce future flood hazard beyond what changes in precipitation extremes alone would suggest. In addition, it can be expected that the nonlinear relationship between flood intensity and impacts (*41*, *43*) means that modest precipitation reductions yield disproportionately large reductions in damage and casualties. Consequently, attributing only the flood hazard, as done here, likely underestimates climate change’s effect on flood impacts.

Our findings contrast with mixed signals in long-term trend analyses of floods for this region. Decreasing trends in annual runoff maxima along the Austrian-Czech border and increasing trends in northern Czechia have been detected in observations, with the mountain ranges between Czechia and Poland marking a transition zone (*6*). Similarly, multimodel projections for a world 1.5°C warmer than preindustrial conditions show only moderate changes in flood magnitude with large uncertainties (*44*). This apparent inconsistency likely reflects the difference between analyzing trends in annual maxima and highly conditioned attribution of individual extreme events to anthropogenic climate change (*45*). In particular, natural climate variability can lead to strong apparent trends in both precipitation extremes (*46*) and floods over the observational period. Using past observed climate as a counterfactual does not allow for disentangling a forced signal related to thermodynamic changes from internal climate variability and other drivers of flood changes (*8*). In contrast, our counterfactual is constructed using observed dynamics but preindustrial greenhouse gas concentrations, isolating the anthropogenic thermodynamic contribution.

Prior end-to-end probabilistic flood event attribution studies have established both the feasibility of coupling climate and hydrological models for attribution and the substantial uncertainties that arise (*47*–*50*). These uncertainties stem in part from the need to constrain preindustrial boundary conditions, such as sea surface temperature, and from the dominance of natural variability. Furthermore, a key limitation in those studies is the need for pooling of events driven by different large-scale circulation patterns, a consequence of characterizing a class of events rather than conditioning on the specific observed synoptic situation (*14*). Our conditional storyline approach (*8*, *51*) addresses both limitations: By fixing atmospheric circulation to that of the observed event, factual and counterfactual simulations differ only in thermodynamic forcing, dynamically distinct event types are not mixed, and the isolated flood magnitude change can be attributed to the thermodynamic contribution of anthropogenic climate change while ignoring other potential confounding effects related to natural variability and circulation changes (*14*).

The substantial differences between CESM2 and AWI-CM-1 storylines—particularly the 9.3% versus 6.4% precipitation reduction in a counterfactual climate and differing spatial patterns of the precipitation and flood peaks—illustrate climate model structural uncertainty. However, this range should not be interpreted as formal uncertainty bounds. Rather, it provides physical insights into sensitivity to model formulation and nudging techniques.

Both models consistently indicate an increase in the precipitation associated with Storm Boris due to anthropogenic climate change, lending confidence to our key findings despite quantitative differences. In addition, the amplified flood-peak increase relative to precipitation is robust across both hydrological models and both climate models.

Several limitations warrant consideration. First, our counterfactual hydrological simulations apply climate change signals from the nudged climate models only during the event period, neglecting altered catchment preconditioning through antecedent soil moisture and snowpack. Our approach also excludes human-induced changes to land use and water management (*14*, *17*), where adaptation has substantially reduced flood-related risks across Europe (*52*). Second, this stratiform precipitation event was particularly suited for our approach using coarse-resolution climate models with parameterized convection, which struggle with convection-driven extremes (*24*, *53*, *54*). Third, storyline attribution constrains atmospheric dynamics, intentionally excluding potential dynamical circulation changes that could modify event likelihood and intensity, for instance via mesoscale convective systems (*55*). Our method quantifies the conditional magnitude change—how much more precipitation and flooding was generated by Storm Boris given that it occurred under the observed circulation patterns—rather than likelihood changes. Fourth, the nudged climate models do not simulate potential climate-change-induced spatial shifts of precipitation below the coarse resolution (∼1°). Small catchments are particularly sensitive to where precipitation falls, and such shifts could, in principle, alter flood hazard independently of changes in total precipitation intensity. Because the factual hydrological simulations are driven by meteorological observations, the present-day flood hazard is not affected by such climate model limitations. However, the attribution of flood hazard changes arising from spatial shifts of precipitation may be incomplete. Such shifts are not apparent in the counterfactual–factual precipitation differences for Storm Boris (Fig. 1, C and D), and our primary metric, relative change in peak runoff at affected locations, is not designed to detect them. Furthermore, spatial shifts in extreme precipitation are highly sensitive to small perturbations in atmospheric conditions, such that even very similar circulation patterns can produce different precipitation footprints, and their attribution is therefore expected to vary substantially across climate models. This further motivates multimodel ensemble approaches for nudged climate model simulations and is a key advantage of emerging kilometer-scale models (see future work below) (*56*, *57*). Last, hydrological model limitations include unrepresented water management infrastructure and simplified routing schemes, although consistency between EARLS and the mHM increases confidence in our conclusions.

Future work could address several of these limitations. Emerging high-resolution nudged storyline simulations, such as those from the IFS-FESOM model at a 9-km resolution (*56*, *57*) or dynamical downscaling of the existing nudged climate simulations (*58*), allow a more detailed representation of mesoscale processes and are close to the meteorological forcing resolution required for direct use in hydrological modeling without the delta approach. Incorporating changes in precipitation, temperature, and evapotranspiration before the flood event into catchment preconditioning would provide a more complete picture of how flood changes are modulated by antecedent soil moisture and snowpack (*17*). More broadly, different attribution methods are suited to different scientific questions (*59*); for events where convective dynamics play a dominant role, complementary approaches such as forecast-based attribution or pseudo–global warming methods may be better suited than nudged climate model simulations. Last, extending this framework to multiple events would allow systematic quantification of climate change impacts on extreme floods beyond single-event case studies, enabling more robust and generalizable conclusions.

Despite these caveats, our results demonstrate that flood hazard attribution requires explicit hydrological modeling rather than precipitation analysis alone. The identified 2.1- to 2.9-fold amplification of flood intensity relative to precipitation changes has direct implications for flood risk assessment and climate-related loss attribution (*60*) under an expected further intensification of precipitation extremes as modest thermodynamic intensification of extreme precipitation events can trigger disproportionate increases in flood hazards.

## MATERIALS AND METHODS

### Observational data, event definition, and statistical analysis

We make use of daily precipitation and minimum, maximum, and mean temperature data over Europe from 1950 to 2024 at 0.1° resolution from the observation-based gridded E-OBS dataset (version 31.0e) (*29*). We define the event duration as 12 to 16 September 2024 (Fig. 1A) following the observed storm duration (*26*, *27*) and previous attribution studies of the same event (*12*, *13*). For analysis and attribution of the flood, we only consider runoff peaks. The period for identifying flood peaks extends to 25 September 2024 to account for delayed flood peaks in large catchments. We consider the spatial extent ranging from 12°E to 20°E and 47°N to 52.5°N to include the footprint of heavy precipitation events and documented impacts (*2*, *28*).

We further use daily runoff observations from national data providers for Czechia (*61*) and Poland (*36*, *62*) to evaluate the hydrological simulations (Fig. 2 and figs. S8 and S9).

To select catchments (EARLS) and grid cells (mHM) for analysis, we fit a stationary generalized extreme value (GEV) distribution to annual maxima from E-OBS–driven (factual) hydrological simulations (see below). We use data from 1951 to 2023 (73 maxima), excluding 1950 for model warm-up and 2024 to avoid bias from the analyzed event (*63*). Only locations for which the flood peak related to Storm Boris has a return period of at least 25 years are included in the analysis, yielding 484 catchments (EARLS) and 377 grid cells (mHM).

For attribution of precipitation and flood peaks, we use the factual climate and hydrological model simulations as reference as the factual event is what has been experienced and thus has higher relevance for comparison (*35*). Factual hydrological simulations also have higher confidence as they are derived directly from meteorological observations without the added uncertainty of the climate change signal removed for the counterfactual simulations. This choice ensures consistent baselines for relative changes in flood peaks (Figs. 3 and 4), avoiding skewed attribution results. For AWI-CM-1 ensemble means (Figs. 2 to 4), we estimate flood peaks for all 25 counterfactuals individually before averaging. For precipitation (Fig. 1D), we average precipitation changes (Eq. 1).

### Hydrological models and factual runoff simulations

We use two structurally different hydrological models to simulate rainfall-runoff relationships. The first is a lumped machine learning model from the EARLS based on the NeuralHydrology framework (*30*, *31*, *64*) trained for daily runoff reconstruction. The second is the physics-based gridded mHM (*37*–*39*, *65*).

The EARLS model uses a long short-term memory (LSTM) neural network architecture for rainfall-runoff modeling. As a lumped model, EARLS requires aggregation of all inputs at the basin level, treating each catchment as a single homogeneous unit. The model uses four dynamic meteorological inputs: daily precipitation, minimum temperature, maximum temperature, and mean temperature. For each simulated day of runoff, the model uses a 365-day sequence of meteorological inputs to capture long-term temporal dependencies.

We use the trained model that has been used to reconstruct daily runoff over 70 years for over 10,000 European catchments (*30*). The model was trained and validated on >5000 catchments from the EStreams dataset (*36*), showing robust performance across diverse European hydrological conditions. To capture catchment-specific characteristics, EARLS incorporates 13 static attributes aggregated from HydroATLAS (*66*, *67*). These attributes include: catchment area, average elevation, average slopes, average stream gradient, average long-term air temperature, minimum long-term air temperature, maximum long-term air temperature, global aridity index, global climate moisture index, average fraction of sand, average fraction of clay, average fraction of silt, and average organic carbon content. This combination of dynamic meteorological forcing and static physiographic characteristics enables the model to capture both temporal variability and spatial heterogeneity in hydrological response. EARLS reproduces the observed 2024 flood peaks (see above how flood peaks are estimated) during Storm Boris relatively well, with correlation coefficients across catchments exceeding 0.7 (fig. S8A) confirming the good overall model performance in the study region (fig. S8B). However, EARLS overestimates the spatial extent of flooding, classifying a larger number of catchments as experiencing extreme events than observed (fig. S9).

The mHM operates at a spatial resolution of 0.125° for both meteorological input processing and routing calculations. The model implements a state-of-the-art multiscale parameter regionalization technique (*37*, *68*), which allows for parameter transfer across scales and regions while maintaining physical consistency. The mHM is initialized with its default parameters and simulates from 1950 to 2024, to ensure adequate spin-up of state variables before the analysis period, for both the factual and the counterfactual hydrological simulations. All simulations of Storm Boris are started from the same restart file on 1 January 2024. The model output *Qrouted* is used for flood peak analyses.

Meteorological forcing data are obtained from the E-OBS dataset (*29*) regridded to 0.125°, with potential evapotranspiration estimated using the Hargreaves-Samani method (*69*), which requires only temperature data as input. The model’s morphological inputs comprise a digital elevation model from the US Geological Survey (*70*), soil properties from SoilGrids (*71*), land cover data from the European Space Agency (*72*), and leaf area index climatology from NASA’s Global Inventory Modeling and Mapping Studies (*73*). Direct comparison with gauge observations is not feasible for the mHM due to its coarse routing resolution.

Neither EARLS’ lumped catchments nor the mHM’s grid cells are spatially independent. EARLS uses nested catchments, whereas the mHM accounts for grid cells throughout the routing network.

### Nudged climate model simulations

We use atmospheric circulation nudging in climate model simulations using the climate models CESM2 and AWI-CM-1 to isolate thermodynamic effects of anthropogenic climate change by constraining atmospheric dynamics under different climate forcing scenarios. Both models use nudging to constrain horizontal winds to ERA5 reanalysis (*74*). This approach enables simulation of observed historical events (fig. S1) and isolates thermodynamic climate change effects from more uncertain dynamical changes (*75*). We perform factual simulations with historical greenhouse gas and aerosol concentrations under Shared Socioeconomic Pathway scenario SSP3-7.0 and counterfactual simulations with preindustrial concentrations.

CESM2 (*32*) is run with prescribed sea surface temperatures where the observed values are fitted to match the preindustrial conditions using a pattern filtering method (*33*). Horizontal winds above 700 hPa are nudged using grid-point nudging to values from ERA5 at 3-hourly time steps while allowing the atmospheric planetary boundary layer to develop freely. Previous experiments showed that this strong constraint renders all ensemble members virtually identical; thus, we use single ensemble members for factual and counterfactual simulations.

For AWI-CM-1 (*76*–*78*), spectral nudging (*79*) of the vorticity and divergence is applied in all vertical levels and wavenumbers, whereas, like in CESM2, all other parameters (including temperature, moisture, clouds, and the ocean) are allowed to develop freely. Sea surface temperatures are likewise unconstrained. In this configuration, the nudging strength is also strong, with a relaxation timescale of 1 hour, which leads to minimal ensemble spread because short-term internal variability is not allowed to develop. Five ensemble members are used for both the factual and counterfactual simulations.

### Counterfactual hydrological simulations

We construct counterfactual simulations by adjusting observed meteorological data with differences from paired factual and counterfactual climate model simulations. We use this, rather than using the climate model output directly, because the climate models feature strong biases even in the nudged setting and lack sufficient spatial resolution to capture localized precipitation extremes, which are crucial for simulating floods with the hydrological models. For CESM2, we obtain a single counterfactual simulation as only a single factual and a single counterfactual run are available. For AWI-CM-1, we create 25 counterfactual simulations from pairwise differences from 5 factual and 5 counterfactual model runs.

For precipitation, we calculate the relative difference between counterfactual and factual simulations for each grid cell as

$$\Delta_{\text{precip}}=\frac{\sum_{\text{event duration}}\;P_{\text{counterfactual}}}{\sum_{\text{event duration}}\;P_{\text{factual}}}\;\text{for}\;P_{\text{factual}},P_{\text{counterfactual}}>\varepsilon$$ (1)

where *P*_factual_ and *P*_counterfactual_ represent precipitation in factual and counterfactual simulations, respectively, and ε = 10 mm prevents unrealistic relative differences when minimal precipitation occurs in either simulation. We then multiply this factor by observed daily precipitation *P*_obs_ to obtain the counterfactual event precipitation

$$P_{\text{counterfactual}}=P_{\text{obs}}\times \Delta_{\text{precip}}$$ (2)

For daily average, minimum, and maximum temperature, we use absolute differences of the mean temperature over the event duration instead

$$\begin{array}{cc} \Delta_{\text{temp}} & =\bar{T_{\text{counterfactual}}}-\bar{T_{\text{factual}}}\\ T_{\text{counterfactual}} & =T_{\text{obs}}+\Delta_{\text{temp}} \end{array}$$ (3)
