# Supplementary material for: Evidence from 2024 Storm Boris indicates anthropogenic climate change amplifies floods more than extreme precipitation
Source: Sci Adv. 2026 Oct 28;12(44):eaef7703. doi: 10.1126/sciadv.aef7703 (PMC13715698; doi:10.1126/sciadv.aef7703)
Supplement: Supplementary file 1 [file sciadv.aef7703_sm.pdf]

Science Advances
AAAS

Supplementary Materials for

Evidence from 2024 Storm Boris indicates anthropogenic climate change amplifies floods more than extreme precipitation

Peter Miersch *et al.*

Corresponding author: Peter Miersch, peter.miersch@ufz.de

Sci. Adv. **12**, eaef7703 (2026)
DOI: 10.1126/sciadv.aef7703

This PDF file includes:

Figs. S1 to S9

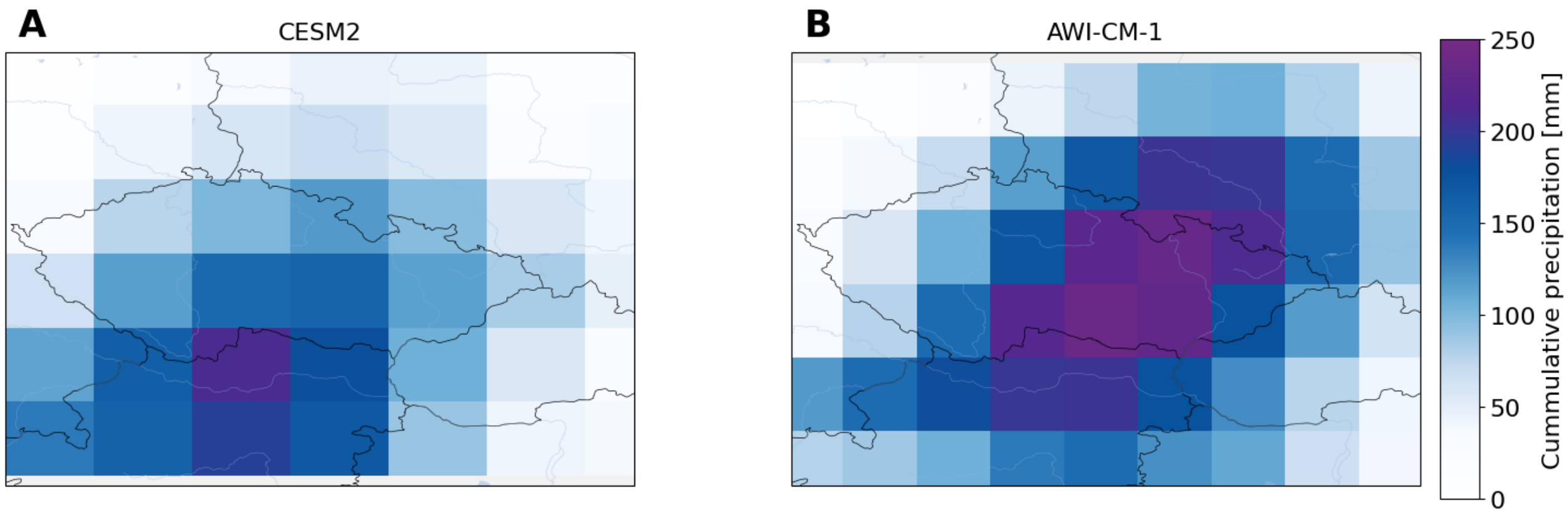

Figure S1: **Simulated event precipitation of nudged climate models for factual emissions.** The event region used is 12°E - 20°E, 47°N, 52.5°N with event duration from 12 to 16 September 2024 (same as in Fig. 1A). (**A**) Cumulative precipitation over the event duration simulated by CESM2. Spatial average: 82 mm, maximum: 209 mm. Compared to observed precipitation (Fig. 1A), CESM2 simulations capture extreme precipitation over Austria well and underestimate precipitation over the border region between Poland and Czechia. (**B**) Same as (A) but for ensemble mean of AWI-CM-1. Spatial average: 116 mm, maximum: 238 mm. Compared to observed precipitation, AWI-CM-1 simulations capture extreme precipitation over both Austria and border region between Poland and Czechia well but overestimate precipitation over Czechia.

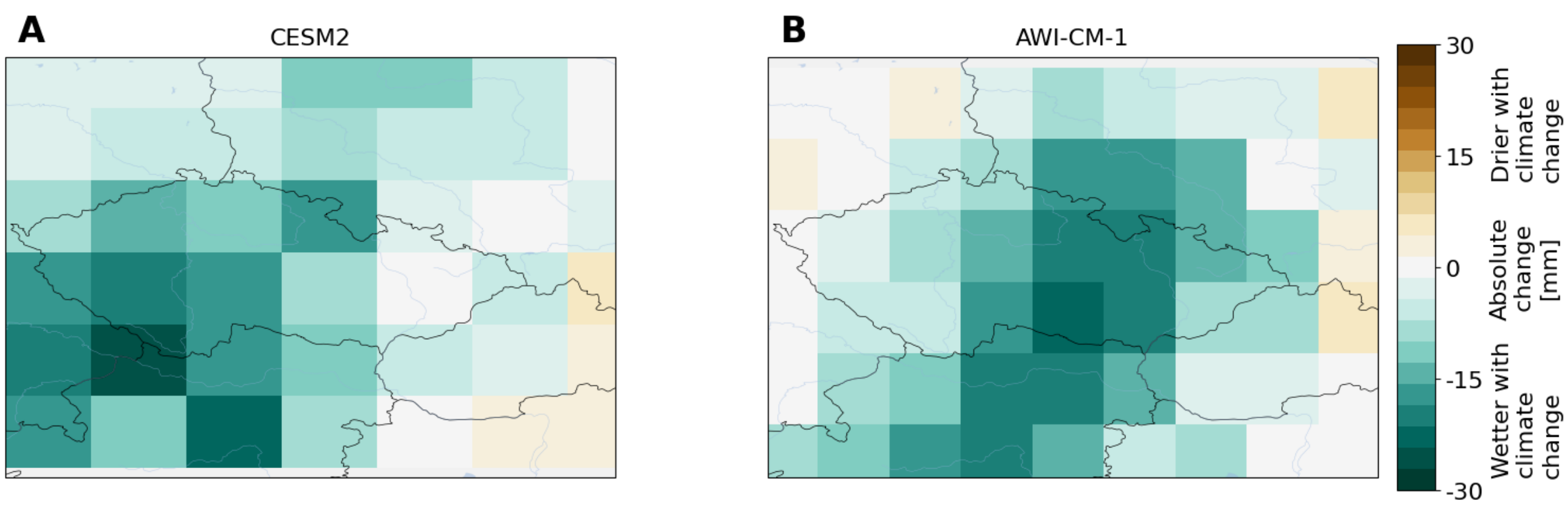

Figure S2: **Precipitation extreme amplified by anthropogenic climate change.** Same as Fig. 1 but for absolute precipitation changes. (**A**) Simulated precipitation change between preindustrial and 2024 greenhouse gas concentrations for CESM2 (mean: −7.6 mm over event region). (**B**) Same as (A) for AWI-CM-1 ensemble mean (mean: −7.4 mm over event region).

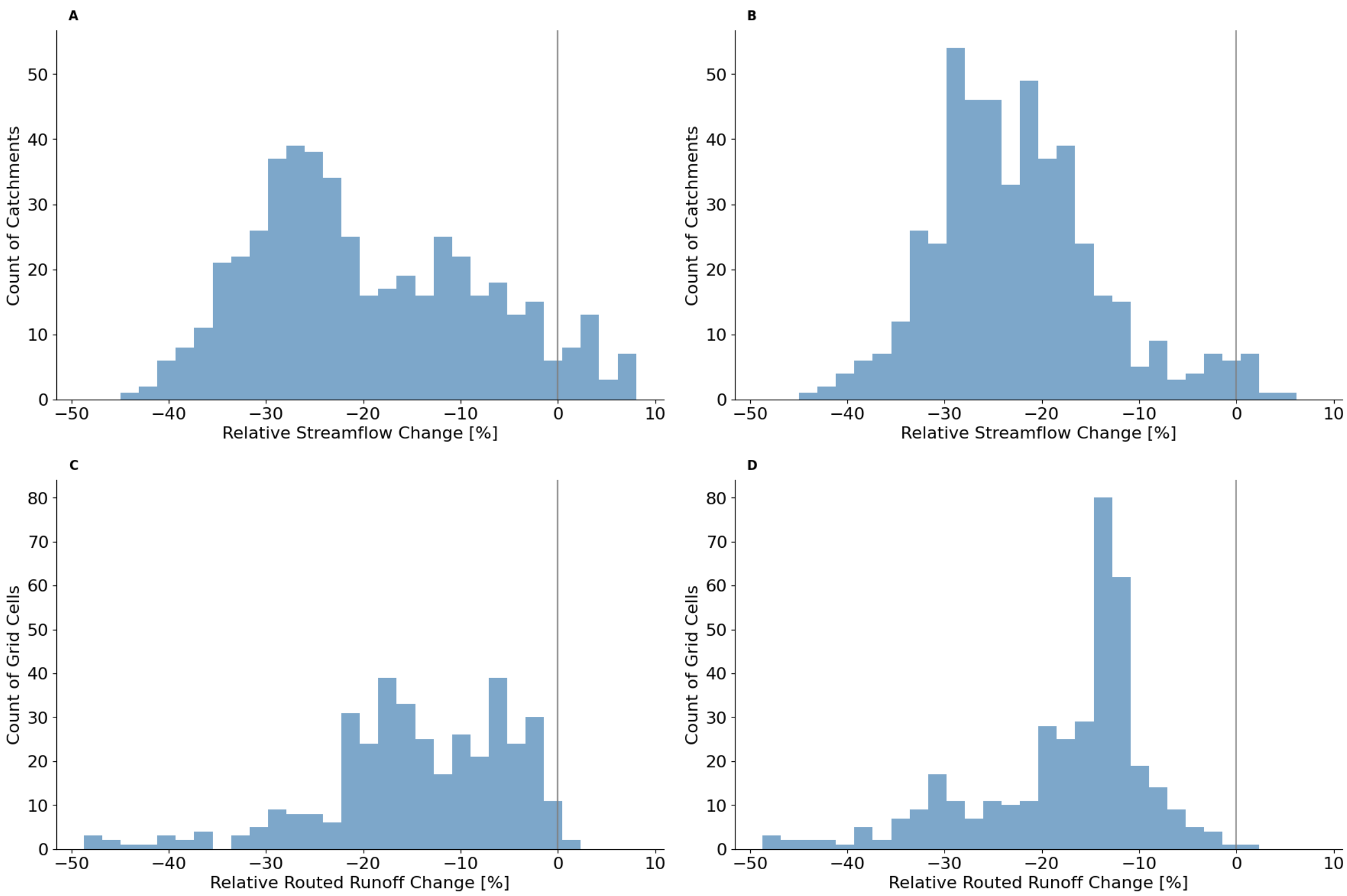

Figure S3: **Flooding was intensified across most catchments and grid cells.** Relative changes in peak runoff between counterfactual and factual simulations. Analysis in catchments (EARLS) and gridcells (mHM) with at least 25-year return period of peak in factual simulation, resulting in 484 catchments and 377 grid cells considered. All changes represent reductions in flood peaks that would occur without anthropogenic climate change. (**A**) Relative change of peak runoff for CESM2 counterfactual. Mean reduction 19.7%. (**B**) Ensemble mean of relative change of EARLS peak runoff for AWI-CM-1 counterfactuals. Mean reduction 22.4%. (**C**) Relative change of mHM peak runoff for CESM2 counterfactual. Mean reduction 14.4%. (**D**) Ensemble mean of relative change of mHM peak runoff for AWI-CM-1 counterfactuals. Mean reduction 17.6%.

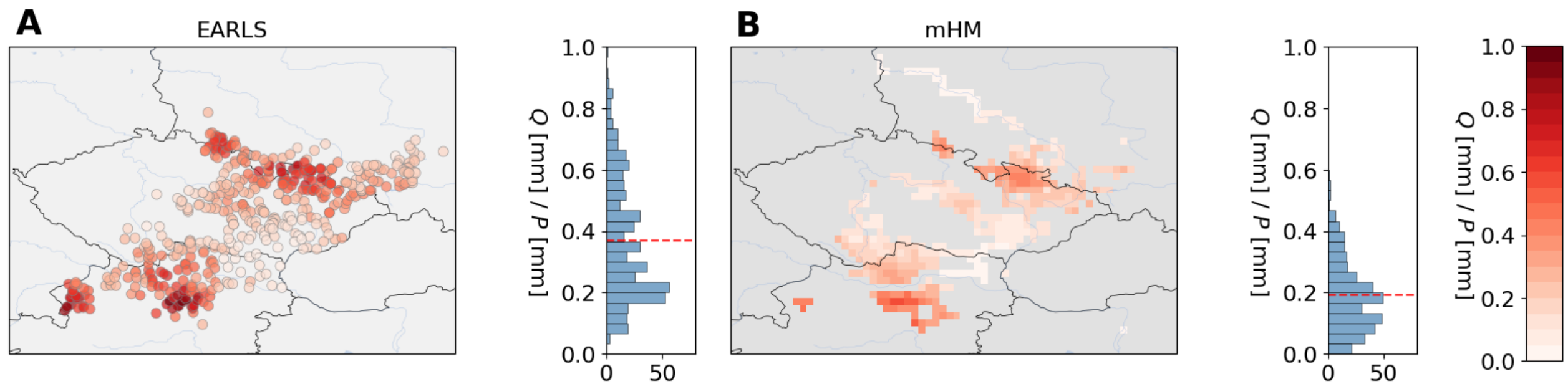

Figure S4: **Runoff coefficient during the event is highest over most impacted regions.** Ratio between runoff Q and event precipitation over the catchment P accumulated over the same event period 12–16 September 2024 (runoff coefficient) for observed meteorological forcings. (**A**) Runoff coefficient simulated with EARLS. Mean coefficient (red line) is 0.38, range 0.06 to 1.02. (**B**) Same as (A) but for simulations with the mHM. Mean coefficient 0.19, range 0.02 to 0.57. Highest coefficients in both models over the regions most impacted by the floods in Lower Austria and the border region between Czechia and Poland. Overall, simulations with the mHM indicate lower runoff coefficients than simulations with EARLS.

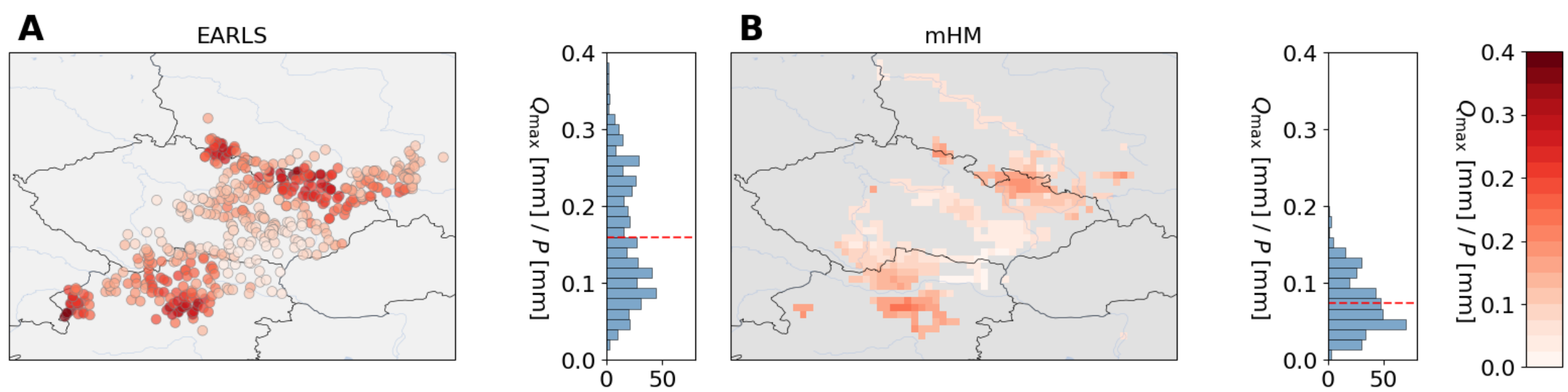

Figure S5: **Peak-runoff coefficient is highest over most impacted regions.** Ratio between the runoff peak Q_{max} and the accumulated event precipitation over the catchment P (peak runoff coefficient) for observed meteorological forcings. (**A**) Peak runoff coefficient simulated with EARLS. Mean coefficient (red line) is 0.16, range 0.02 to 0.40. (**B**) Same as (A) but for simulations with the mHM. Mean coefficient 0.07, range 0.02 to 0.19. Highest coefficients in both models over the regions most impacted by the floods in Lower Austria and the border region between Czechia and Poland. Overall, simulations with the mHM indicate lower peak runoff coefficients than simulations with EARLS.

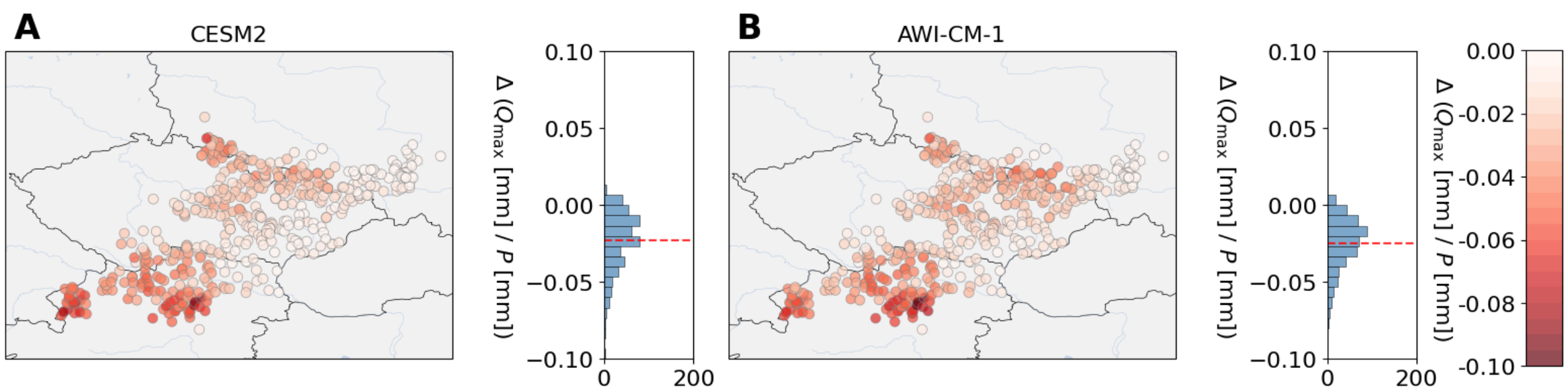

Figure S6: **Peak runoff coefficient was amplified by climate change as simulated with EARLS** Change in the ratio of the runoff peak Q_{max} and the accumulated event precipitation over the catchment P (peak runoff coefficient) between counterfactual and factual meteorological forcings. Negative values indicate that event precipitation was converted less efficiently to a runoff peak in the counterfactual hydrological simulations without climate change. (**A**) Difference in peak runoff coefficient between factual and counterfactuals from CESM2. Mean change (red line) is -0.02, range -0.09 to 0.01. (**B**) Same as (A) but for the ensemble mean of AWI-CM-1. Mean coefficient -0.03, range -0.10 to 0.00. Highest change in peak runoff coefficients in both climate models over the regions most impacted by the floods, especially in Lower Austria and to a lesser extent in the border region between Czechia and Poland. Overall peak runoff coefficient was lower in the counterfactual hydrological simulations without climate change.

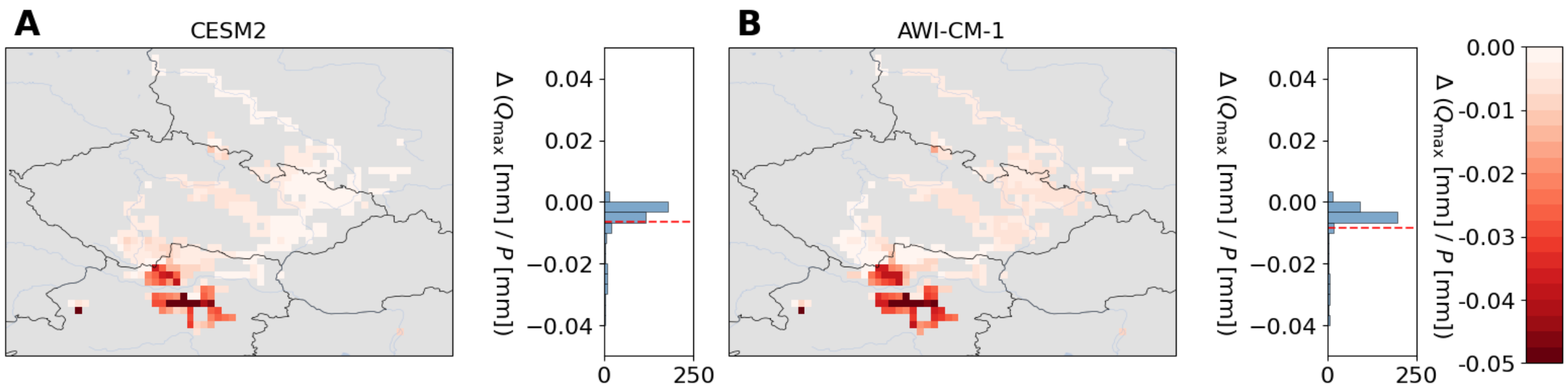

Figure S7: **Peak runoff coefficient was amplified by climate change as simulated with the mHM** Change in the ratio of the runoff peak Q_{max} and the accumulated event precipitation over the catchment P (change in peak runoff coefficient) between counterfactual and factual meteorological forcings. Negative values indicate that event precipitation was converted less efficiently to a runoff peak in the counterfactual hydrological simulations without climate change. (**A**) Difference in rainfall-runoff ratio between factual and counterfactual hydrological simulations with CESM2. Mean change (red line) is -0.007, range -0.078 to 0.003. (**B**) Same as (A) but for the ensemble mean of AWI-CM-1. Mean ratio -0.008, range -0.080 to 0.006. Highest change in peak runoff coefficients in both climate models over flood impacted regions in Lower Austria, with small changes everywhere else. Changes are smaller than simulated with EARLS in most regions. Overall change in peak runoff coefficient was lower in the counterfactual hydrological simulations without climate change. This is in agreement with overall lower peak runoff coefficients in the mHM compared to EARLS in the factual hydrological simulations (fig. S5)

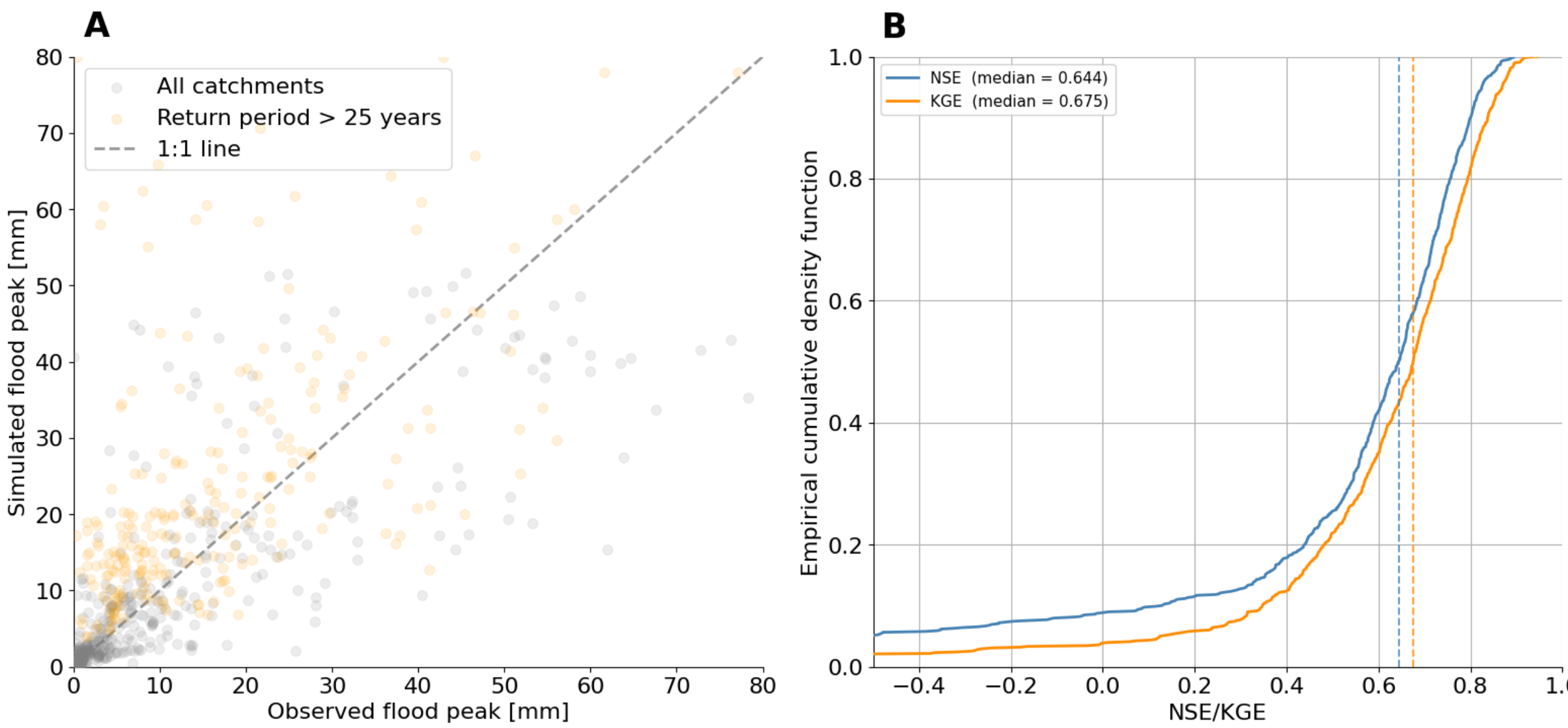

Figure S8: **Hydrological model evaluation for EARLS.** (**A**) Comparison of simulated with observed flood peaks. Observed runoff peaks of the event available for 714 stations (gray), 267 of which are used in this analysis based on a 25-year return period flood (orange) compared to simulated runoff peaks with EARLS. The correlation between observed and simulated peaks is 0.722 for all catchments and 0.718 for catchments that experienced at least a 25-year flood. (**B**) Empirical cumulative density function of Nash-Sutcliffe Efficiency (NSE, blue line) and Kling-Gupta efficiency (KGE, orange line) for 777 stations in the study region with at least 20 years of observational data in EStreams. Median NSE is 0.64 and median KGE is 0.68 (dashed lines).

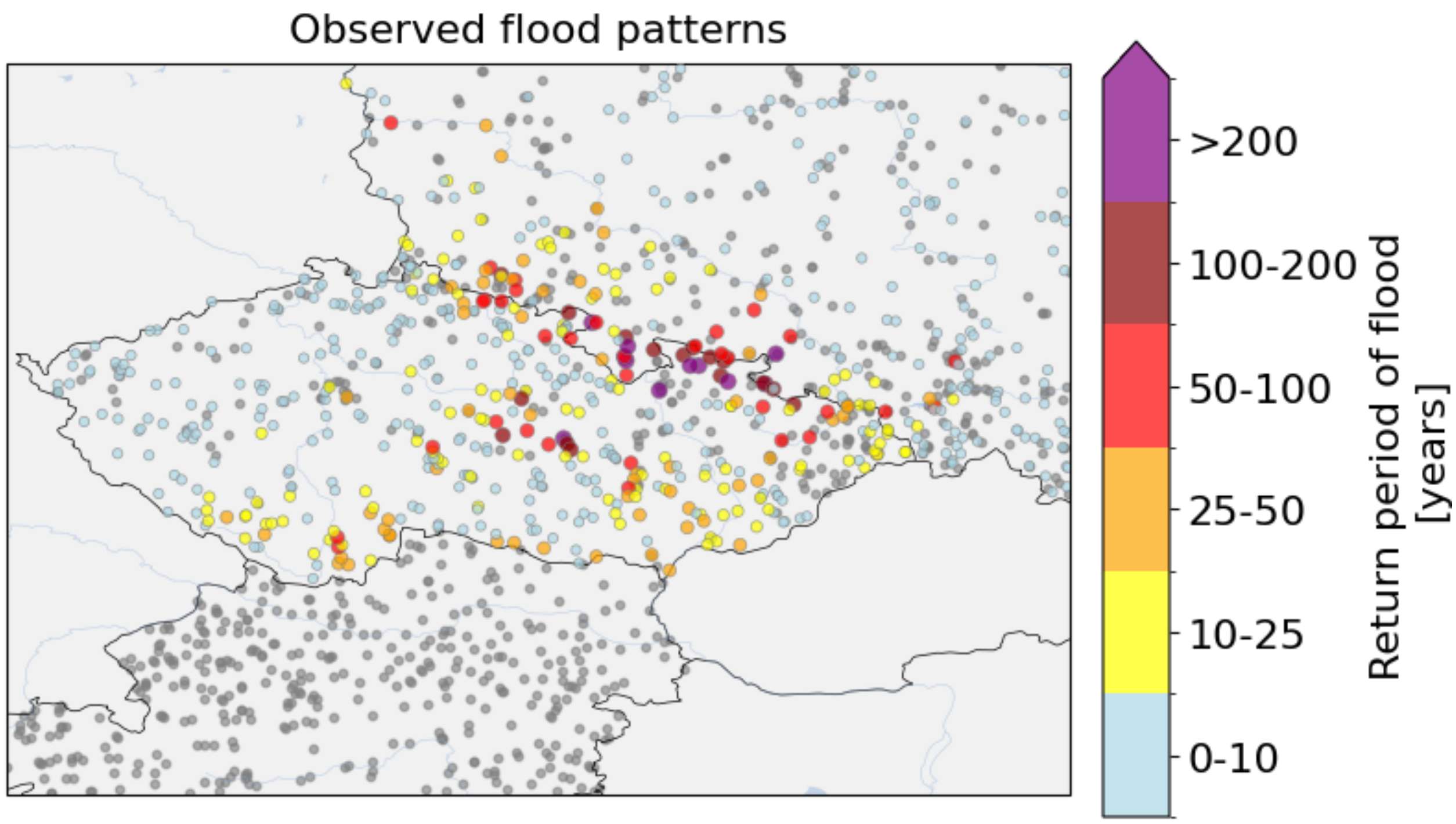

Figure S9: **Observed flood return periods during Storm Boris.** As Fig. 1B, but for observed flood peaks. A stationary generalized extreme value distribution was fitted to observed runoff records from catchments with at least 20 years of data to estimate return periods. Grey dots denote catchments with insufficient or missing observations; no observational data are available for Austrian catchments during Storm Boris. EARLS correctly captures the flooding along the Czech–Polish border but overestimates its spatial extent both along the border and across Czechia.
